# Comparison of the complete genomes of two *Echinochloa* species: barnyard grass and jungle rice

**DOI:** 10.1080/23802359.2017.1361355

**Published:** 2017-08-08

**Authors:** Jeongran Lee, Jin-Won Kim, In-Yong Lee, Jong Hwa Ahn

**Affiliations:** a Department of Agro-food Safety and Crop Protection, National Institute of Agricultural Sciences (NAS), Iseo, Republic of Korea;; b National Instrumentation Center for Environment Management (NICEM), College of Agriculture and Life Sciences, Seoul National University, Seoul, Korea

**Keywords:** Chloroplast genome, *Echinochloa colona*, *Echinochloa crus-galli*

## Abstract

We sequenced and compared the complete chloroplast (cp) genomes of the two *Echinochloa* species; *E. crus-galli* (KT983256) and *E. colona* (KT983255). The size of complete chloroplast genomes of the two species are 139,851 and 139,592 base pairs in length, respectively. They include a pair of inverted repeats (22,640 and 22,618 bp) separated by the small (12,518 and 12,519 bp) and large (82,053 and 81,837 bp) single copy regions. Both chloroplast genomes include a total of 136 genes. Phylogenetic analysis revealed that *E. colona* was diverged between 2.65 and 3.18 million years ago (Mya) from the *E. oryzicola* and *E. crus-galli*.

The cosmopolitan genus, *Echinochloa* consisting of approximately 30–50 species, includes the most nuisant weeds in agricultural fields (Clayton and Renvoize 1999). One of the *Echinochloa* species*, E. colona* is a mostly serious weed in the tropical Asia, Australia and Pacific Islands as well as in the northern part of South America and the Caribbean (Holm et al. 1977). Although *E. colona* has not been reported its seriousness in the temperate zone, it is possible to cause a problem in the temperate zone like the southern part of South Korean rice fields because of global warming. To provide the cpDNA sequence information and insight into the divergence time of the *Echinochloa* species we sequenced the chloroplast genomes of *E. colona* and *E. crus-galli* and analyzed the phylogenetic relationships among several related taxa.

*Echinochloa colona* (PI292598) introduced from US which originally collected from Israel and *E. crus-galli* collected from Shinan, South Korea (35°05’04.6"N 126°13’36.1"E) were kept at National Institute of Agricultural Sciences (Iseo, Korea) and used for Genomic DNA isolation using a Genomic DNA Isolation Kit (NucleoGen, Germany). Library construction and illumina Hiseq 2500 sequencing were conducted at the National Instrumentation Center for Environmental Management (Seoul, Korea). The cp genome assembly was performed using Celera Assembler 6.1 (Celera Genomics, Alameda, CA) and Abyss version 1.3.0 assembler (Simpson et al. 2009) followed by annotation using the web-based program Dual Organellar GenoMe Annotator (Wyman et al. 2004).

The barnyard grass chloroplast genome is 139,851 bp in length and contains a pair of inverted repeats (IRs) of 22,640 bp each, separated by a large and small single copy region (LSC and SSC) of 82,053 bp and 12,518 bp, respectively. The jungle rice chloroplast genome is 139,592 bp in length, with IRs of 22,618 bp each, separated by an LSC of 81,837 bp and an SSC of 12,519 bp. The barnyard grass chloroplast genome is 259 bp longer than that of the jungle rice. The G + C content of both barnyard grass and jungle rice is 38.6% across the whole cpDNA. Gene contents and arrangement are identical in both cpDNAs. There are 88 unique protein-coding genes in both genomes, nine of which are duplicated (*ndh*B, *rpl*2, *rpl*23, *rps*7, *rps*12, *rps*15, *rps*19, *ycf* 1, and *ycf*68) in the IR. Five out of six single-copy protein-coding genes, *rbc*L, *cem*A, *clp*P, *inf*A, and *mat*K, were located in LSC region while *ccs*A was in SSC. The four rRNA genes are contained completely within the IRs. There are 22 unique tRNA genes, of which seven are in the IR, bringing the total number to 40 in the genome. There are 11 unique intron-containing genes; nine genes have a single intron and two genes have two introns.

The phylogenetic tree was constructed using BEAST (Drummond et al. 2012) with Yule process and divergence time of 50–60 million years ago (Mya) between *Oryza sativa* and *Sorghum bicolor* (Ma et al. 2005; Nah et al. 2015) to estimate the divergence time of a total of 15 monocot cp genome sequences. *Typha latifolia* (NC013823) was used as an outgroup. Phylogeny revealed that *Echinochoa* was monophyly. *Echinochloa colona* was diverged from *E. oryzicola* and *E. crus-galli* 2.65–3.18 Mya (Figure 1).

**Figure 1.**
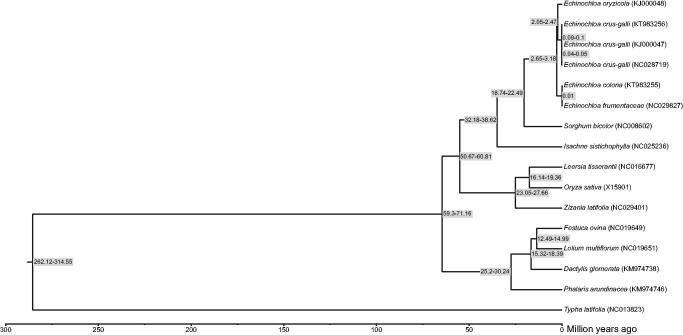
Phylogenetic relationship and divergence time of *Echinochloa colona* and *E. oryzicola*. *Typha latifolia* (NC013823) was included as an outgroup. Numbers in gray boxes indicate the range of estimated divergence time in million years.

## Nucleotide sequence accession numbers

The cp genome sequences were submitted to the GenBank with accession numbers of KT983255 and KT983256.

## References

[CIT0001] Clayton WD, Renvoize SA. 1999 Genera graminum, grasses of the world. London (UK): Royal Botanic Gardens, Kew p. 280–281.

[CIT0002] Drummond AJ, Suchard MA, Xie D, Rambaut A. 2012 Bayesian phylogenetics with BEAUti and the BEAST 1.7. Mol Biol Evol. 29:1969–1973. 22367748 10.1093/molbev/mss075 PMC3408070

[CIT0003] Holm LG, Plucknett DL, Pancho JV, Herberger JP. 1977 The world’s worst weeds: distribution and biology, Honolulu (Hawaii): The University Press of Hawaii p. 41–46.

[CIT0004] Ma J, Sanmiguel P, Lai J, Messing J, Bennetzen JL. 2005 DNA rearrangement in orthologous Orp regions of the maize, rice and sorghum genomes. Genetics. 170:1209–1220. 15834137 10.1534/genetics.105.040915 PMC1451190

[CIT0005] Nah G, Im JH, Kim JW, Kim K, Lim J, Choi AY, Choi IY, Yang TJ, Park TS, Lee D, et al. 2015 The complete chloroplast genomes of three Korean *Echinochloa crus-galli* accessions. Mitochondrial DNA A. 27:4357–4358. 10.3109/19401736.2015.1089499 26466198

[CIT0006] Simpson JT, Wong K, Jackman SD, Schein JE, Jones SJM, Birol I. 2009 ABySS: a parallel assembler for short read sequence data. Genome Res. 19:1117–1123. 19251739 10.1101/gr.089532.108 PMC2694472

[CIT0007] Wyman SK, Jansen RK, Boore JL. 2004 Automatic annotation of organellar genomes with DOGMA. Bioinformatics. 20:3252–3255. 15180927 10.1093/bioinformatics/bth352

